# Factors related to the probability of suffering mental health problems in emergency care professionals

**DOI:** 10.1590/1518-8345.3079-3144

**Published:** 2019-04-29

**Authors:** Silvia Portero de la Cruz, Jesús Cebrino Cruz, Javier Herruzo Cabrera, Manuel Vaquero Abellán

**Affiliations:** 1 Universidad de Córdoba, Facultad de Medicina y Enfermería, Departamento de Enfermería, Córdoba, Andalucía, Espanha; 2 Universidad de Sevilla, Facultad de Medicina, Departamento de Medicina Preventiva y Salud Pública, Sevilla, Andalucía, Espanha; 3 Universidad de Córdoba, Facultad de Ciencias de la Educación, Departamento de Psicología, Córdoba, Andalucía, Espanha

**Keywords:** Adaptation Psychological, Burnout Professional, Nursing, Occupational Health, Mental Health, Emergency Service Hospital, Adaptação Psicológica, Esgotamento Profissional, Enfermagem, Saúde do Trabalhador, Saúde Mental, Serviço Hospitalar de Emergência, Adaptación Psicológica, Agotamiento Profesional, Enfermería, Salud Laboral, Salud Mental, Servicio de Urgencia en Hospital

## Abstract

**Objectives:**

to evaluate the influence of burnout and coping strategies used by health professionals of the hospital emergency service on their mental health status and to determine sociodemographic and labor characteristics.

**Method:**

descriptive cross-sectional study in a sample of 235 nursing professionals and physicians who worked in four hospital emergency services. The Maslach Burnout Inventory, the General Health Questionnaire and the *Inventario Breve de Afrontamiento*-cope 28 were used as data collection instruments and specific and original questionnaires of sociodemographic and labor variables. Descriptive, quantitative and multivariate statistics were applied.

**Results:**

the dimension of depersonalization, avoidance-centered coping and being a physician were related to the presence of somatic symptoms, anxiety, social dysfunction and depression. Increased professional experience was associated with greater social dysfunction among health personnel and increased number of patients was related to depressive symptoms among health professionals.

**Conclusions:**

the dimensions of emotional exhaustion and depersonalization, avoidance-centered coping, being a physician and a daily smoker increase the risk of a psychiatric case. The practice of daily physical exercise is a protective factor.

## Abstract (pt)

**Objetivos:**

avaliar a influência exercida pelo *Burnout* e as estratégias de enfrentamento utilizadas pelo pessoal de saúde do serviço de emergência hospitalar sobre o estado de saúde mental e determinar as características sociodemográficas e laborais.

**Método:**

estudo descritivo transversal em uma amostra de 235 profissionais de enfermagem e médicos que atuaram em quatro serviços de emergência hospitalar. Como instrumentos de coleta de dados foram utilizados questionários originais e específicos de variáveis sociodemográficas e de trabalho, o *Maslach Burnout Inventory*, *General Health Questionnaire* e o *Inventario breve de afrontamiento–COPE 28*. Aplicou-se estatística descritiva, inferencial e multivariante.

**Resultados:**

a dimensão de despersonalização, enfrentamento centrado na evitação e ser médico estiveram relacionados à presença de sintomas somáticos, ansiedade, disfunção social e depressão. O aumento da experiência profissional esteve associado à maior disfunção social do pessoal de saúde e o aumento do número de pacientes foi relacionado à sintomatologia depressiva entre os profissionais de saúde.

**Conclusões:**

as dimensões de exaustão emocional e despersonalização, enfrentamento centrado na evitação, ser médico e consumir tabaco diariamente aumentam o risco de se constituir um caso psiquiátrico. A prática de exercício físico diário é um fator de proteção.

## Abstract (es)

**Objetivos:**

evaluar la influencia que ejerce el *burnout* y las estrategias de afrontamiento empleadas por el personal sanitario del servicio de urgencias hospitalario sobre el estado de salud mental y determinar las características sociodemográficas y laborales relacionadas.

**Método:**

estudio descriptivo transversal en una muestra de 235 profesionales de enfermería y medicina que trabajaban en cuatro servicios de urgencias hospitalarios. Como instrumentos de recogida de datos se utilizaron cuestionarios originales y específicos de variables sociodemográficas y laborales, el *Maslach Burnout Inventory*, el *General Health Questionnaire* y el *Inventario breve de afrontamiento–COPE 28.* Se aplicó estadística descriptiva, inferencial y multivariante.

**Resultados:**

la dimensión de despersonalización, el afrontamiento centrado en la evitación y ser médico estuvieron relacionados con la presencia de síntomas somáticos, de ansiedad, disfunción social y depresión. El aumento de la experiencia profesional estuvo asociado a una mayor disfunción social del personal sanitario y el incremento del número de pacientes se relacionó con la sintomatología depresiva entre los profesionales sanitarios.

**Conclusiones:**

las dimensiones de agotamiento emocional y despersonalización, el afrontamiento centrado en la evitación, ser médico y consumir tabaco diariamente incrementan el riesgo de constituir un caso psiquiátrico. La práctica de ejercicio físico diario constituye un factor protector.

## Introduction

The need to include mental health in the priorities of public health programs has been recognized for several decades. The promotion of mental health has a special relevance in the work environment, since it is an important factor in the development of physical and mental problems^(^ ^1^ ^)^. In this sense, the number of countries reporting occupational diseases, especially mental changes such as neurosis, paranoia, depression, anxiety, insomnia or fatigue, is growing^(^ ^2^ ^)^.

In the health field, the prevalence of psychiatric morbidity in health professionals working in the hospital emergency service is 36.8%^(^ ^3^ ^)^, in an often stressful work environment that implies, among others, the management of diagnostic and therapeutic uncertainties, which can produce burnout^(^ ^4^ ^)^.

Burnout is defined as a three-dimensional psychological syndrome consisting of emotional exhaustion, depersonalization, and personal fulfillment that occurs in response to chronic work stress^(^ ^5^ ^)^. Emotional exhaustion causes a decrease or loss of emotional resources, depersonalization leads to the development of negative attitudes toward patients, and lack of personal fulfillment is characterized by decreased feelings of competence and achievement at work^(^ ^6^ ^)^.

The incidence of burnout has increased in recent years and numerous studies have been carried out with the aim of establishing the causes and factors associated with it^(^ ^2^ ^)^. Thus, a growing number of studies indicate that women increase the risk of suffering burnout based on the double presence of women at home and at work, lower wages, or greater at work^(^ ^2^ ^,^ ^7^ ^)^.

The highest rates of burnout correspond to professionals working in the emergency department compared to the other specialties^(^ ^8^ ^–^ ^9^ ^)^. In the nursing team, high levels of burnout produce a higher incidence of musculoskeletal disorders, occupational injuries, absenteeism, job dissatisfaction, as well as abuse of alcohol and other drugs^(^ ^10^ ^)^. In addition, they negatively affect, among others, the quality of care received by patients^(^ ^11^ ^)^ and the mental health of nursing professionals, which can cause depression, anxiety, low self-esteem, or feelings of guilt^(^ ^12^ ^)^.

One factor that may moderate the negative impact of burnout on the physical and psychological well-being of health professionals is their coping style^(^ ^13^ ^)^. Coping is the process by which the individual, by cognitive, emotional or behavioral efforts, manages, controls, or reduces the demands of the individual-environment that have been assessed as stressful^(^ ^14^ ^)^. There are two styles of great coping. On the one hand, there is the active or adaptive coping, used when stressful conditions are evaluated by the individual as subject to change, modification or elimination using strategies such as the search for problem solving or positive re-reading. On the other hand, there is the passive or maladaptive coping used when stress conditions are assessed as permanent or non-modifiable. This type of coping includes behavioral strategies of escape or evasion, and behavioral and cognitive distancing or selective attention in order to reduce emotional problems^(^ ^15^ ^)^. The use of a particular strategy depends on the previous level of psychological and family adaptation of the individual, and on available social resources^(^ ^16^ ^)^.

Several studies have focused on analyzing the binomials of burnout-mental health^(^ ^17^ ^)^ or burnout-coping strategies^(^ ^13^ ^)^, or mental health-coping strategies^(^ ^18^ ^)^ in emergency care professionals. Both individual and organizational consequences may arise from burnout and the response generated by professionals in similar situations or conditions is different. In view of this, it is necessary to deepen the relationships between burnout, coping strategies, and mental health together as a preliminary step towards the implementation of prevention and intervention programs that are useful in the promotion and implementation of new coping strategies with a comprehensive approach at the individual, social and organizational level and that allow adequate levels of mental health in the emergency hospital services.

The objectives of this study are to evaluate the influence exercised by burnout and coping strategies used by emergency care professionals in their mental health status and to determine the sociodemographic and labor characteristics.

## Method

A descriptive, cross-sectional and multicenter study was carried out from March to December 2016, in the emergency department of four hospitals in the Autonomous Community of Andalusia (Spain). Two hospitals were public (Hospital 1: 1302 beds with 214 medical and nursing professionals in the emergency service, Hospital 2: 880 beds with 167 medical and nursing professionals in the emergency service) and two were private hospitals (Hospital 3: 154 beds with 42 medical and nursing professionals in the emergency service, Hospital 4: 220 beds with 55 medical and nursing professionals in the emergency service). Data on the total number of beds and health personnel working in the emergency rooms were provided to the researchers by the nursing managers of the different hospitals, who were chosen by incidental sampling. The study population consisted of all medical and nursing professionals from the emergency services of these hospitals.

Active workers were included during data collection and those whose time of work in the hospital emergency service was equal to or greater than one year. Intern specialists and health professionals treated with antidepressants and/or anxiolytics were excluded.

A sample of 235 subjects with a confidence level of 95%, an absolute precision of 4% and a psychiatric morbidity of 25.50% was set^(^ ^19^ ^)^, assuming that the population was 478 individuals. The sample size was calculated using the Epidat 4.0 program and was based on presuming the lowest advantageous prevalence.

Three validated, calibrated and self-administered questionnaires were used, as well as another one that included sociodemographic variables: gender (male, female), age (years), marital status (married, single, divorced, widowed) daily practice of physical exercise (yes, no) and daily tobacco consumption (yes, no), as well as labor variables: professional category (physician, nurse), type of contract (fixed, provisional, eventual), time of work in the hospital emergency service (years), professional experience (years), and number of patients attended daily.

The burnout evaluation was performed through the Maslach Burnout Inventory (MBI) in its adapted version for the Spanish language^(^ ^20^ ^)^. The MBI evaluates three dimensions of burnout: emotional exhaustion, depersonalization, and personal fulfillment. The questionnaire consists of 22 items, with a Likert-type response format, in which professionals indicate the frequency of stated situations from zero (never) to six points (daily).

To determine the score of each dimension, the scores of the items belonging to each dimension are added. The emotional exhaustion dimension is composed of nine items, with a minimum total score of zero point and maximum of 54 points. The depersonalization dimension is composed of five items, the total score ranging from zero to 30 points. The personal fulfillment dimension consists of eight items and the total score varies between 0 and 48 points. The higher the score obtained in the dimensions of emotional exhaustion and depersonalization, the worse the level of burnout, while the interpretation of the personal fulfillment dimension is the reverse. Dimensions were categorized into low, medium and high level, taking into account the following cutoff points: emotional fatigue (<18 points: low, 19-26 points: medium, > 27 points: high), depersonalization (<5 points: low, 6-9 points: medium, > 10 points: high) and personal achievement (<33 points: low, 34-39 points: medium, > 40 points: high)^(^ ^21^ ^)^. Low levels of the dimensions emotional exhaustion and depersonalization and high personal fulfillment are indicative of absence of burnout. On the other hand, high burnout is characterized by high levels of emotional exhaustion and depersonalization and low levels of personal fulfillment. Average level of burnout is found in the rest of the cases.

The General Health Questionnaire (GHQ-28) was applied in its validated version to the Spanish language^(^ ^22^ ^)^ to assess the subjects' mental health. It consists of 28 items divided into four subscales of seven items: somatic symptoms (psychological somatic symptoms, such as tiredness, fatigue, headaches or general malaise), anxiety (difficulty falling asleep, frequent arousals, irritability or psychic tension), social dysfunction (inability to make decisions or to perform organized development of work, leading to worse daily functioning) and depression (mood-related symptoms that even include suicidal ideation). The subscales represent dimensions of the symptomatology, therefore do not correspond to psychiatric diagnoses. Each item is evaluated using a scale of four possible responses, ranging from zero (less than normal) to three (much more than normal). For the evaluation of the results, the bimodal response scale was used, in which different positions share a score. In this way, possible errors due to the choice of extreme or central responses are avoided. Thus, zero is scored if either of the first two options is answered, and one is scored if either of the latter two is answered (0011). For each of the subscales, the score can vary from zero (absence of symptoms) to seven (maximum frequency of symptoms).

Scores ≤6 show absence of metal changes, while scores equal to 7 are indicative of the presence of a probable psychiatric case.

The subjects' coping was measured with the *Inventario breve de afrontamiento* - COPE 28, adapted to the Spanish language^(^ ^23^ ^)^. The questionnaire was used in a dispositional manner, and the subjects responded by referring to how they deal with stressful situations. The questionnaire consists of 28 items with four response options on a Likert scale ranging from zero (I do not do any of this) to three (I do this very often). The items are grouped into fourteen scales of two items each. These scales represent three types of coping: problem-centered coping (consisting of active coping scales, planning and finding instrumental support, with a total of six items), emotion-focused coping (consisting of emotional support search scales, positive reinterpretation, denial, acceptance, religion, and humor, with a total of 12 items) and avoidance-focused coping (consisting of scales of self-distraction, venting, behavioral withdrawal, substance use, self-incrimination, with a total of 10 items). In order to obtain the total score of the coping types, the scores of the items that make up each of them are added and divided by the number of items that make up each type of coping. Thus, the types of coping vary between 0 and 3 points. High scores indicate greater use of the strategy.

The questionnaires were handed out to each participant at the beginning of the work shift and returned directly to the researchers at the end of the work shift. Together with the questionnaires, an informative letter was attached, detailing the objectives of the study, the form of collaboration of the subjects, as well as the voluntary and anonymous nature of the said participation, and an explicit request for collaboration in which the health professional gave his/her consent to participate in the study.

The study was approved by the Research Ethics Committee of the Reina Sofia University Hospital of Córdoba, Spain (registration number 249, reference 3050) and by the managers of the emergency service of participating centers, in accordance with the principles of Helsinki.

For the analysis of the qualitative variables, a frequency table was used to measure the mean and standard deviation. To establish possible relationships between the qualitative variables, the Chi-square test or the Fisher's exact test were use, and the Student's t-test and analysis of variance were applied for the quantitative variables. The normal distribution of the data was verified by the Kolmogorov-Smirnov test. In cases where the normality hypotheses were not met, non-parametric tests (Mann-Whitney U or Kruskal-Wallis) were applied. For the correlation of variables, the Pearson or Spearman correlation coefficient was calculated according to the applicability conditions. Four multiple linear regression models were performed using the reverse variable selection method, one for each GHQ-28 subscale. The dependent variables of each model were somatic symptoms, anxiety, social dysfunction and depression. In addition, a binary logistic regression model was performed, whose dependent variable was the presence/absence of probable psychiatric cases detected by GHQ-28. The independent variables included in all models were gender, age, marital status, daily practice of physical activity, daily tobacco consumption, professional category, type of contract, length of service in the hospital, professional experience, number of patients attended daily, emotional exhaustion, depersonalization, personal fulfillment, problem-centered coping, emotion-centered coping, and avoidance-centered coping. In each model, by means of the Wald statistic, the variables with p ≥ 0.15 were eliminated one by one. The continuous variables scale was evaluated by the Box-Tidwell test. The possible interactions between all the variables were studied. The variables with significance greater than 0.05 were studied as possible confounding factors, considering them as if the percentage variation of the coefficients was greater than 15%. In the multiple linear regression models, the residue normality was verified by the Kolmogorov-Smirnov test. We did not consider the existence of collinearity problems among the independent variables if the factor of increase in the variance was less than or equal to 10. As a diagnostic test of extreme cases, the analysis of student residues was used. The adjusted coefficient of determination R^2^ was used to evaluate the adequacy of adjustment. The gross and adjusted values of the ß coefficient were determined. In the binary logistic regression model, the likelihood ratio test and Nagelkerke R^2^ were used to determine the quality of adjustment. The area under the Receiver Operating Characteristic (ROC) curve was calculated to determine which explanatory variables are most associated with the probability of establishing a psychiatric case. Gross and adjusted Odds Ratio (OR) values were determined. Values p <0.05 were considered significant. For statistical analysis, the G-Stat program, version 2, was used.

## Results

A total of 235 professionals participated in the study. The female population constituted the predominant population, with 76.2% (Table 1). The mean age was 48.3 (8) years. Most participants were single (61.7%) and 48.9% reported using tobacco daily. In addition, 54.5% reported practicing daily exercise. The labor variables showed that the majority of the sample consisted of nursing professionals (72.8%) and that the most frequent type of contract was the fixed one (66.4%). In addition, the average professional experience was 22.7 (8.7) years, and the mean time of work at the emergency ward was 12 (9.4) years. The mean number of patients attended daily was 102.4 (63.2), and 55.7% of the professionals had an average level of emotional exhaustion and increased depersonalization (48.9%) and personal fulfillment (54.9%), which indicates that the level of burnout was average. Concerning symptoms related to mental health, professionals presented a higher frequency of anxiety symptoms, with a mean score of 2.5 (1.9) points. Likewise, problem-focused coping and emotion-focused coping strategies were the most commonly used, with mean scores of 1.5 (0.5) and 1.3 (0.4), respectively. In addition, one-third of the professionals (32.3%) were likely to suffer from psychiatric disorder.

**Table 1:** Description of the sociodemographic-labor characteristics and dimensions-subscales of the Maslach Burnout Inventory, General Health Questionnaire and *Inventario Breve de Afrontamiento* - COPE 28 in health professionals of the hospital emergency service. Andalusia, Spain, 2016

Variable		Frequency (Percentage) n=235
Sex		
	Male	56 (23.8)
	Female	179 (76.2)
Daily physical exercise	
	Yes	128 (54.5)
	No	107 (45.5)
Daily consumption of tobacco	
	Yes	115 (48.9)
	No	120 (51.1)
Professional category	
	Physician	64 (27.2)
	Nurse	171 (72.8)
Type of contract	
	Fixed	156 (66.4)
	Provisional	58 (24.7)
	Eventual	21 (8.9)
Emotional exhaustion	
	High	46 (19.6)
	Average	131 (55.7)
	Low	58 (24.7)
Depersonalization	
	High	115 (48.9)
	Average	42 (17.9)
	Low	78 (33.2)
Personal fulfillment	
	High	129 (54.9)
	Average	45 (19.1)
	Low	61 (26)
Psychiatric case	
	Yes	76 (32.3)
	No	159 (67.7)
**Variable**		**Mean (Standard deviation)**
Time of work (years)		12 (9.4)
Professional experience (years)		22.7 (8.7)
Patients seen daily		102.4 (63.2)
Maslach Burnout Inventory	
	Emotional exhaustion (points)	17.4 (11.3)
	Depersonalization (points)	8.7 (6.1)
	Personal achievement (points)	37.9 (8.4)
General Health Questionnaire	
	Somatic symptoms	2.4 (2)
	Anxiety	2.5 (1.9)
Social dysfunction		2.3 (1.8)
	Depression	2 (2)
*Inventario breve de afrontamiento - COPE 28*	
	Problem-centered coping (points)	1.5 (0.5)
	Emotion-centered coping (points)	1.3 (0.4)
	Avoidance-centered coping (points)	1.1 (0.5)

The bivariate analysis (Table 2) found a relationship between avoidance-centered coping and increased frequency of somatic symptoms (r = 0.5), anxiety (r = 0.6), social dysfunction (r = 0.5), and depression (r = 0.6) (p <0.001). Other variables related to the four subscales of the symptoms were, on the one hand, the number of patients (somatic symptoms: r = 0.2, anxiety: r = 0.3, social dysfunction: r = 0.2 and depression: r = 0.3) (p <0.001) and, on the other hand, the medical staff in relation to nursing professionals (difference of the somatic symptom score = 0.7, anxiety = 1.1, social dysfunction = 0.8 and depression = 1.2) (p <0.001). In addition, mean emotional exhaustion was higher among psychiatric cases compared to non-psychiatric cases (18.7 vs. 15.1 points, p <0.01).

**Table 2:** Relationship between the dimensions and subscales of the Maslach Burnout Inventory, *Inventario breve de afrontamiento*- COPE 28 and sociodemographic-labor variables, and the subscales of the General Health Questionnaire in emergency care professionals. Andalusia, Spain, 2016

Variable		SS* (points) n=235	A† (points) n=235	SD‡ (points) n=235	D§ (points) n=235	PC|| (points) n=235	No PC|| (points) n=235
		r Pearson¶	r Pearson¶	r Pearson¶	r Pearson¶	Mean (Standard deviation)	Mean (Standard deviation)
EE** (points)		0.1	0.2	0.2††	0.1	18.7(11.8)	15.1(10.1)‡‡
DP§§ (points)		0.01	0.1	0.04	0.04	8.7 (5.9)	8.7 (6.6)
PF|||| (points)		0.03	-0.03	-0.03	0.1	37.8 (8.5)	38.1 (8.2)
PCC¶¶ (points)		-0.1	-0.1	-0.3††	-0.2‡‡	1.5 (0.5)	1.6 (0.5)
ECC*** (points)		0.2	0.1	0.1	0.2	1.3 (0.4)	1.2 (0.4)
ACC†† † (points)		0.5††	0.6††	0.5††	0.6††	1.3 (0.4)	0.8 (0.3)
Age (years)		0.1	0.1	0.1	0.1	49.1 (7.3)	46.7 (8.9)‡‡
Time of work (years)		-0.1	-0.04	-0.1	-0.1	11.5 (9.6)	13.1 (9.1)
Professional experience (years)		0.1	0.1	0.1‡‡	0.1	23.3 (9)	21.4 (7.8)
Patients seen daily		0.2††	0.3††	0.2††	0.3††	55.8(23.2)	43.3 (248)
		**Mean (Standard deviation)**	**Mean (Standard deviation)**	**Mean (Standard deviation)**	**Mean (Standard deviation)**	**n (%)**	**n (%)**
Sex							
	Male	2.2 (2)	2.2 (1.9)	2.1 (1.8)	2 (2)	34 (60.7)	22 (39.3)
	Female	2.5 (1.9)	2.6 (1.9)	2.4 (1.8)	2 (2)	120 (67)	59 (33)
Marital status							
	Married	1.9 (1.9)	2.1(2.1)	1.9 (1.9)	1.4 (2)	27 (50.9)	26 (49.1)
	Single	2.5 (2)	2.6 (1.9)	2.4 (1.8)	2.1 (2)	98 (67.6)	47 (32.4)
	Separated/divorced	2.8 (1.9)	2.7 (1.7)	2.8 (1.6)	2.4 (1.9)	22 (81.5)	5 (18.5)
	Widowed	2.2 (2.2)	2.8 (2)	2.7 (2.2)	2.2 (1.9)	7 (7)	3 (30)
Daily physical exercise							
	Yes	2 (1.8)	2.2 (2)	2 (1.8)	1.7 (2)	94 (73.4)	34 (26.6)
	No	2.7 (2)††	2.7 (1.9)	2.6(1.8)‡‡	2.3 (1.9)	60 (56.1)	47 (43.9)‡‡
Daily consumption of tobacco							
	Yes	2.6 (1.9)	2.6 (1.9)	2.4 (1.9)	2.3 (2)	83 (72.2)	32 (27.8)
	No	2.2 (2)	2.3 (2)	2.3 (1.8)	1.7 (1.9)	71 (59.2)	49 (40.8)‡‡
Professional category							
	Physician	2.9 (1.8)	3.3 (1.6)	2.9 (1.7)	2.9 (1.7)	54 (84.4)	10 (15.6)
	Nurse	2.2 (2)††	2.2 (2)††	2.1(1.8)††	1.7 (2)††	100 (58.5)	71 (41.5)‡‡
Type of contract							
	Fixed	2.5 (1.9)	2.6 (1.9)	2.5 (1.8)	2.1 (2)	106 (67.9)	50 (32.1)
	Provisional	2 (2.1)	1.9 (2)	1.9 (1.8)	1.7(2)‡‡ ‡‡‡	24 (52.2)	32 (47.8)
	Eventual	2.6(2.1)	3 (2.1)	2.3 (1.8)	2 (2)	14 (60.9)	9 (39.1)

* SS: Somatic symptoms;

† A: Anxiety.

‡ SD: Social dysfunction;

§ D: Depression.

|| PC: Psychiatric case;

¶ Direct regression: somatic symptoms = 0.1 + 2 × avoidance-centered coping; Somatic symptoms = 1.5 + 0.02 × patients seen daily; Anxiety = −0.1 + 2.3 × avoidance-centered coping; Anxiety = 1.4 + 0.02 × patients seen daily; Social dysfunction = 3.8-0.9 × problem-centered coping; Social dysfunction = −0.02 + 2 × avoidance-centered coping; Social dysfunction = 1.6 + 0.03 × work experience; Social dysfunction = 1.7 + 0.01 × patients seen daily; Depression = 3.1 - 0.7 × problem-centered coping; Depression = −0.8 + 2.5 × avoidance-centered coping; Depression = 0.8 + 0.02 × patients seen daily;

** EE: Emotional exhaustion;

†† p value <0.001.

‡‡ p value <0.01;

§§ DP: Depersonalization;

|||| PF: Personal fulfillment;

¶¶ PCC: problem-centered coping;

*** ECC: emotion-centered coping;

†† † ACC: avoidance-centered coping;

‡‡‡ Significance obtained by the analysis of variance and Scheffé's method for later comparisons: significant versus eventual difference

The conditions of the multiple linear regression model of residual normality were met, no collinearity was found between the independent variables and no extreme value was found for subscales of somatic symptoms and anxiety. The frequency of somatic symptoms increased 0.02 points for each increase in the depersonalization dimension score (p = 0.01), 2 points for each increase of the avoidance-centered coping score (p = 0, 0001) and 0.3 points among the medical staff in relation to nursing professionals (p = 0.02). The frequency of anxiety symptoms increased by 0.1 points for each increase in the depersonalization dimension score (p = 0.01), doubling for each increase in the avoidance-centered coping score (p = 0.001) and increased 0.7 points among the medical staff in relation to nursing professionals (p = 0.001) (Table 3).

**Table 3:** Factors related to the subscales of somatic symptoms and anxiety of the General Health Questionnaire in emergency care professionals. Andalusia, Spain, 2016

Variable		Somatic symptoms*				Anxiety†			
		Gross estimate		Adjusted estimate‡		Gross estimate		Adjusted estimate‡	
		Coef ß	P-value	Coef ß	P-value	Coef ß	P-value	Coef ß	P-value
EE§ (points)		0.02	0.1			0.03	0.1		
DP|| (points)		0.1	0.03	0.02	0.01	0.01	0.02	0.1	0.01
PF¶ (points)		-0.04	0.9			-0.2	0.2		
PCC** (points)		-0.3	0.2			-0.2	0.4		
ECC†† (points)		0.2	0.6			-0.4	0.2		
ACC‡‡ (points)		1.9	0.001	2	0.0001	2.3	0.002	2	0.001
Age (years)		0.03	0.2			-0.3	0.7		
Time of work (years)		0.6	0.7			-0.4	0.1		
Professional experience (years)		0.02	0.2			0.01	0.5		
Patients seen daily		0.2	0.4			0.2	0.1		
Sex									
	Female	1 (Ref.)	0.7			1 (Ref.)	0.4		
	Male	0.1				0.2			
Marital status									
	Married	1 (Ref.)				1 (Ref.)			
	Single	0.3	0.3			0.3	0.8		
	Separated/divorced	0.8	0.9			0.1	0.8		
	Widowed	0.6	0.3			0.6	0.4		
Daily physical exercise									
	Yes	1 (Ref.)				1 (Ref.)			
	No	0.7	0.1			0.3	0.1		
Daily consumption of tobacco									
	Yes	1 (Ref.)				1(Ref.)			
	No	0.3	0.2			0.2	0.4		
Professional category									
	Physician	0.4		0.3		0.8		0.7	
	Nurse	(1 Ref.)	0.01	(1 Ref.)	0.02	(1 Ref.)	0.01	(1 Ref.)	0.001
Type of contract									
	Fixed	1 (Ref.)				1 (Ref.)			
	Provisional	0.2	0.3			0.4	0.2		
	Eventual	0.3	0.5			0.7	0.1		

* Coefficient of determination adjusted for the subscale of somatic symptoms = 23.8%, F = 25.4, p <0.001;

† Coefficient of determination adjusted for the anxiety subscale = 34.9%, F = 21.9, p<0.001;

‡ Adjustment variables: age and sex;

§ EE: Emotional exhaustion;

|| DP: Depersonalization;

¶ PF: Personal fulfillment;

** PCC: problem-centered coping;

†† ECC: emotion-centered coping;

‡‡ ACC: avoidance-centered coping

The conditions of the multiple linear regression model of residual normality, non-collinearity between the independent variables and non-existence of extreme values for the subscales of social dysfunction and depression were met. The frequency of symptoms of social dysfunction decreased by 1 point for each increase in frequency of problem-focused coping score (p = 0.002), increased by 0.5 points for each year of work experience (p = 0, 04), increased by 2 points for each increase in the avoidance-centered coping score (p = 0.001), and increased by 0.5 points among the medical staff in relation to nursing professionals (p = 0.03). In addition, the frequency of depressive symptoms increased by 0.2 points for each increase in avoidance-focused coping frequency (p = 0.03), increasing by 0.4 points for each patient attended daily by professionals (p = 0.04) and doubled among the medical staff in relation to nursing professionals (p = 0.001) (Table 4).

**Table 4:** Factors related to subscales of social dysfunction and depression of the General Health Questionnaire in emergency care professionals. Andalusia, Spain, 2016

Variable		Social dysfunction*				Depression†			
		Gross estimate		Adjusted estimate‡		Gross estimate		Adjusted estimate‡	
		Coef ß	P-value	Coef ß	P-value	Coef ß	P-value	Coef ß	P-value
AE§ (points)		0.02	0.1			0.04	0.3		
DP|| (points)		0.1	0.2			0.1	0.1		
PF¶ (points)		-0.01	0.4			-0.1	0.6		
PCC** (points)		-1	0.001	-1	0.002	-0.8	0.5		
ECC†† (points)		0.1	0.8			0.2	0.4		
ACC‡‡(points)		2	0.004	2	0.001	0.2	0.01	0.2	0.03
Age (years)		-0.02	0.2			-0.03	0.1		
Time of work (years)		-0.02	0.2			-0.2	0.7		
Professional experience (years)		0.3	0.04	0.5	0.04	0.03	0.1		
Patients seen daily		0.3	0.3			0.32	0.01	0.4	0.04
Sex									
	Female	1 (Ref.)	0.3			1 (Ref.)	0.4		
	Male	0.2				0.2			
Marital status									
	Married	1 (Ref.)				1 (Ref.)			
	Single	0.03	0.9			0.2	0.5		
	Separated/divorced	0.1	0.9			0.4	0.5		
	Widowed	0.4	0.3			0.4	0.3		
Daily physical exercise									
	Yes	0.4	0.1			0.4	0.1		
	No	1 (Ref.)				1 (Ref.)			
Daily consumption of tobacco									
	Yes	1 (Ref.)				1 (Ref.)			
	No	-0.1	0.03			-0.5	0.3		
Professional category									
	Physician	0.4		0.5		1.3		2.1	
	Nurse	1 (Ref.)	0.02	(1 Ref.)	0.03	(1 Ref.)	0.01	(1 Ref.)	0.001
Type of contract									
	Fixed	1 (Ref.)				1 (Ref.)			
	Provisional	-0.2	0.4			0.02	0.9		
	Eventual	0.2	0.5			0.2	0.6		

* Coefficient of determination adjusted for the social dysfunction subscale = 36.7%, F = 23.6, p <0.001;

† Coefficient of determination adjusted for the subscale of depression = 40.6%, F = 33.1, p <0.001;

‡ Adjustment variables: age and sex;

§ EE: Emotional exhaustion;

|| DP: Depersonalization;

¶ PF: Personal fulfillment;

** PCC: problem-centered coping;

†† ECC: emotion-centered coping;

‡‡ ACC: avoidance-centered coping

The risk of having a probable psychiatric case was determined by increased emotional exhaustion (adjusted OR = 1.07, p = 0.01), depersonalization (adjusted OR = 1.87, p = 0.001), avoidance-centered coping (OR = 4.37, p = 0.001), daily consumption of tobacco (adjusted OR = 2.38, p = 0.02) and being a physician (adjusted OR = 1.30, p = 0.01), while daily practice of exercise was a protective factor (adjusted OR = 0.30, p = 0.01) (Table 5).

**Table 5:** Factors related to the likelihood of a psychiatric case through the use of the General Health Questionnaire in emergency care professionals. Andalusia, Spain, 2016

Variable*		Gross Odds Ratio (CI† 95%)	P-value	Adjusted Odds Ratio ‡,§ (CI† 95%)	P-value
EE|| (points)		1.03 (1.01 – 1.06)	0.02	1.07 (1.02 – 1.11)	0.01
DP¶ (points)		1.97 (1.92 – 1.99)	0.01	1.87 (1.80 – 1.94)	0.001
PF** (points)		1.14 (0.95 – 1.23)	0.19		
PCC†† (points)		0.61 (0.34 – 1.06)	0.08		
ECC‡‡ (points)		2.09 (0.98 – 4.44)	0.06		
ACC§§ (points)		3.98 (3.91 – 4.87)	0.01	4.37 (3.51 – 6.78)	0.001
Age (years)		1.04 (0.87 – 1.07)	0.17		
Time of work (years)		0.98 (0.95 – 1)	0.20		
Professional experience (years)		1.02 (0.99 – 1.06)	0.12		
Patients seen daily		1.02 (0.95 – 1.03)	0.07		
Sex					
	Female	1.32 (0.71 – 2.45)	0.39	
	Male	1 (Ref.)			
Marital status					
	Married	1 (Ref.)			
	Single	0.28 (0.16 – 1.20)	0.33		
	Separated/divorced	0.84 (0.67 – 2.35)	0.28		
	Widowed	0.63 (0.24 – 1.51)	0.16		
Daily physical exercise					
	Yes	0.16 (0.10 – 0.74)		0.30 (0.22 – 0.44)	
	No	1 (Ref.)	0.01	1 (Ref.)	0.01
Daily consumption of tobacco					
	Yes	1.79 (1.03 – 3.09)	0.04	2.38 (1.14 – 5)	0.02
	No	1 (Ref.)		1 (Ref.)	
Professional category					
	Physician	1.26 (1.12 – 1.55)	0.01	1.30 (1.12 – 1.78)	0.01
	Nurse	1 (Ref.)		1 (Ref.)	
Type of contract					
	Fixed	1 (Ref.)			
	Provisional	0.20 (0.12 – 1.43)	0.53		
	Eventual	0.31 (0.25 – 1.05)	0.39		

* Dependent variable: presence/absence of probable psychiatric cases detected by the General Health Questionnaire (≤6 points: normal probability, no case and 7 points: probable psychiatric case);

† Confidence interval;

‡ Adjustment variables: age and sex;

§ Probability ratio test = 35.2, R2 of Nagelkerke = 0.6, Area under the ROC curve = 0.9, p <0.001;

|| EE: Emotional exhaustion;

¶ DP: Depersonalization;

** PF: Personal fulfillment;

†† PCC: problem-centered coping;

‡‡ ECC: emotion-centered coping;

§§ ACC: avoidance-centered coping

## Discussion

In this study, on the one hand, the variables related to the presence of somatic symptoms in health professionals were on the dimension of depersonalization, avoidance-centered coping and being a physician. These last two variables were also related to the symptomatology of anxiety, social dysfunction and depression. In addition, the depersonalization dimension was related to anxiety; professional experience and problem-centered coping were related to social dysfunction and to the number of patients treated daily with depressive symptomatology. On the other hand, the risk of being a psychiatric case was determined by the level of emotional exhaustion, depersonalization, the use of avoidance-centered coping, daily consumption of tobacco and being a physician, while daily physical exercise was a protective factor.

The mean burnout scores placed the sample at a low level of emotional exhaustion and a means of depersonalization and personal fulfillment. These findings are similar to those obtained in previous studies^(^ ^13^ ^,^ ^24^ ^)^. Although the level of burnout was average, according to a recent study^(^ ^9^ ^)^, the highest prevalence rate of burnout was found in the hospital emergency ward (71%).

The proportion of possible psychiatric cases among the health professionals studied was 32.3%, similar to that obtained in another study^(^ ^3^ ^)^. The problem-centered coping was the most commonly used strategy, which could explain the relative low morbidity found. This finding is consistent with that obtained in another study^(^ ^25^ ^)^.

The dimensions of emotional exhaustion and depersonalization increased the risk of being a psychiatric case among health professionals. Depersonalization, moreover, was related to the presence of somatic symptoms and anxiety. However, the dimension of personal fulfillment has been left out of these relationships. Some authors have highlighted the relationship between emotional exhaustion and depersonalization and the appearance of symptoms related to a worse level of mental health^(^ ^26^ ^–^ ^27^ ^)^, having the dimension of emotional exhaustion as the strongest link^(^ ^27^ ^–^ ^28^ ^)^.

The avoidance-centered coping strategy was related to somatic symptomatology, anxiety, social and depressive dysfunction in professionals. In addition, it increased the risk of being a psychiatric case. These findings are in agreement with the results of another study^(^ ^18^ ^)^. However, this type of coping may be the best option for emergency personnel working outside the hospital environment, especially when the event occurs, in order to avoid emotional involvement^(^ ^29^ ^)^. In addition, a negative relationship was found between the use of the problem-focused coping strategy and social dysfunction. Evidence indicates that there is a significant relationship between a good level of mental health and the use of the problem-focused coping strategy. On the other hand, and more specifically, those who have trouble maintaining or initiating a relationship with others (socialization) tend to experience inadequate emotional growth, loneliness, depression and do not get used to using types of active coping^(^ ^30^ ^)^.

Regarding the sociodemographic and labor characteristics related to mental health status, it was found that medical personnel had a higher risk of being a psychiatric case than nursing professionals. In addition, being a physician was related to the four subscales that make up the GHQ-28 (somatic symptoms, anxiety, social dysfunction and depression). Although it is true that nursing is considered one of the most stressful occupations, which may increase the risk of suffering psychiatric disorders^(^ ^31^ ^)^, several studies conclude that in the emergency service, medical personnel are more likely to report justified psychological adverse outcomes based on their responsibility^(^ ^32^ ^)^, while nursing professionals point to a higher level of job dissatisfaction^(^ ^8^ ^,^ ^24^ ^)^. Although nursing professionals do not present mental changes, they appear to be influenced by coping strategies. In this sense, the person-environment adjustment can moderate the perceived stress, the work-family conflict and the level of mental health. In addition, clinical supervision seems to mediate stress and mental health in this group^(^ ^33^ ^)^.

The present study found a positive relationship between professional experience and the social dysfunction scale of GHQ-28, contrary to that found in other research^(^ ^3^ ^)^. This finding can be explained by two facts. On the one hand, the evidence shows the positive relationship between work experience and the burnout level^(^ ^34^ ^)^. On the other hand, the presence of symptoms associated with worse daily functioning (ability to concentrate or enjoy activities) could be related to an increase in the burnout level^(^ ^35^ ^)^.

In addition, it was found that the daily increase in the number of patients seen in the emergency department was related to the greater presence of depressive symptoms manifested by professionals. The literature has shown that increasing numbers of patients and of professionals' overload increase the risk of depressive symptoms among health professionals^(^ ^36^ ^)^. Similarly, in emergencies, the constant flow of patients cared for by nursing staff is considered a factor associated with burnout in this group^(^ ^37^ ^)^.

The risk of being a psychiatric case was reduced in professionals who practiced daily exercise and increased among those who consumed tobacco daily. Evidence indicates that physical exercise attenuates the anxious response to emotional stimuli^(^ ^38^ ^)^ and that depression and anxiety rates are higher among nicotine dependents^(^ ^39^ ^)^.

The limitations of this study include a possible selection bias in the study population, since it is subject to the professionals' degree of interest in participating in the study. In addition, the results point to associations, but do not allow establishing cause and effect relationships. Although Andalusia is a representative area of the health system, it would also be interesting to consider professionals from other geographical locations.

This work highlights the need to continue with lines of research that use more complex projects to determine the role played by burnout and the passive coping in the mental health of health professionals. Thus, health care weaknesses could be determined and mental health control increased, thereby reducing the negative consequences that result from an inadequate level for the system and the user.

## Conclusion

Nursing professionals do not present mental changes. Increased levels of emotional exhaustion, depersonalization, avoidance-centered coping, being a physician, and consuming tobacco daily increase the risk of becoming a psychiatric case. In turn, the practice of daily physical exercise is a protective factor. The variables positively related to psychological somatic symptomatology and anxiety are the dimension of depersonalization, the use of avoidance-centered coping strategies and the being a physician. Social dysfunction is directly associated with the fact of being a physician, with the use of coping focused on avoidance and with professional experience, and inversely related to the use of problem-centered coping strategies. Finally, the presence of depressive symptoms is related to the use of avoidance-centered coping strategies, being a physician and increased number of patients attended daily.
